# Supplementary material for: Sphingopyxis sp. Strain OPL5, an Isoprene-Degrading Bacterium from the Sphingomonadaceae Family Isolated from Oil Palm Leaves
Source: Microorganisms. 2020 Oct 10;8(10):1557. doi: 10.3390/microorganisms8101557 (PMC7600658; doi:10.3390/microorganisms8101557)
Supplement: Supplementary file 1 [file microorganisms-08-01557-s001.pdf]

Supplementary Figures

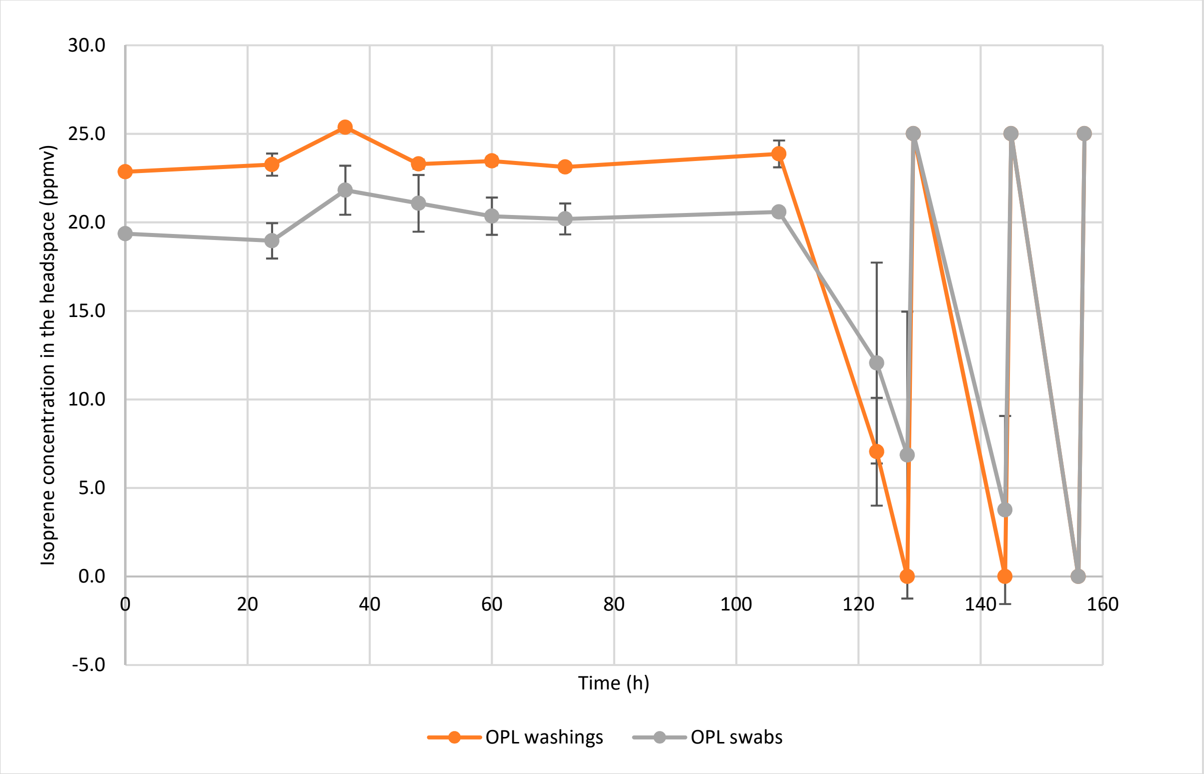

Figure S1. Isoprene consumption in microcosms from oil palm leaf (OPL) washings and swabs. Leaf microcosms were enriched and replenished with ^{12}C-isoprene several times. Data points show the mean of triplicates and error bars show the standard deviation.

A

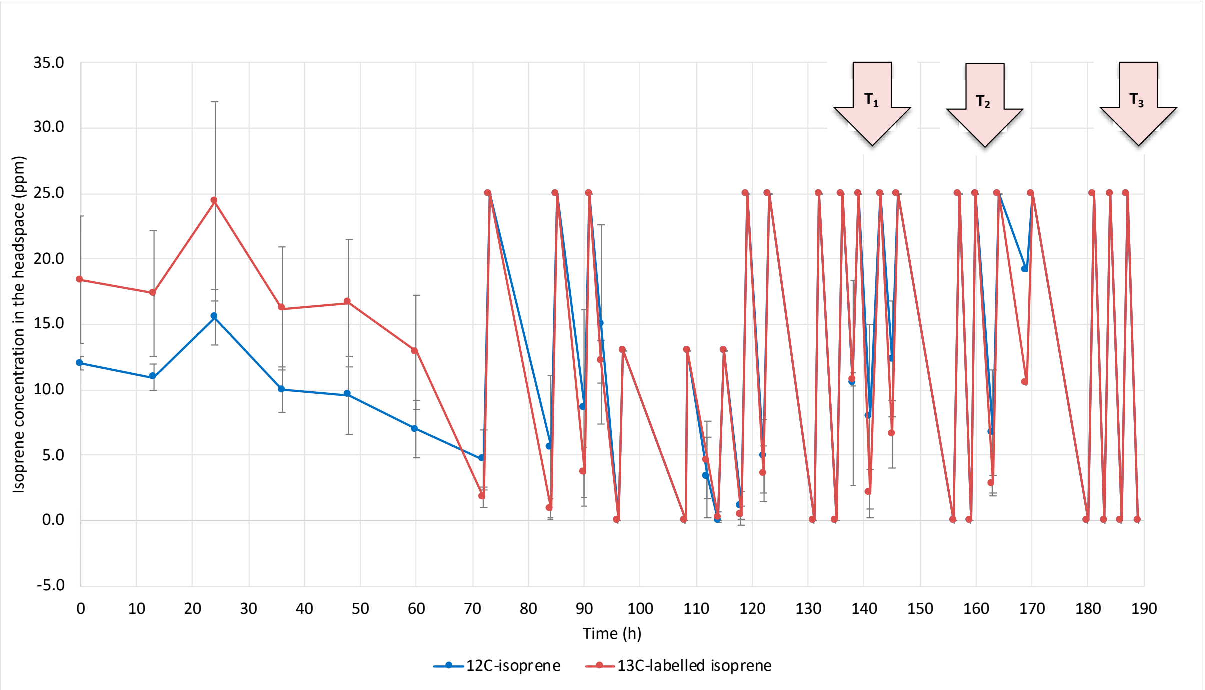

B

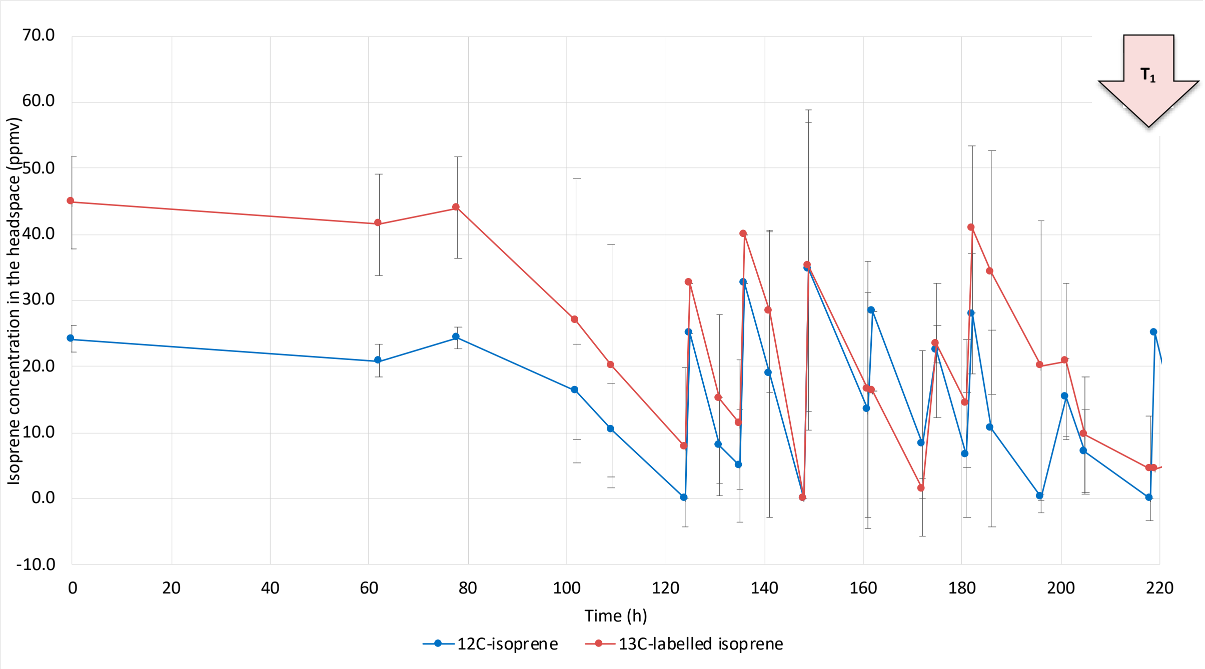

Figure S2. Isoprene consumption in microcosms used in DNA-SIP experiments with oil palm soil (A) and leaf washings (B).

Soil (A) and leaf washings (B) microcosms were enriched and replenished with ^{12}C- or ^{13}C-labelled isoprene. Data points show the mean of triplicates and error bars show the standard deviation. B) Leaf

washings enrichment are shown up to 220 h (T_1) because of variation observed in the isoprene consumption between replicates.

A

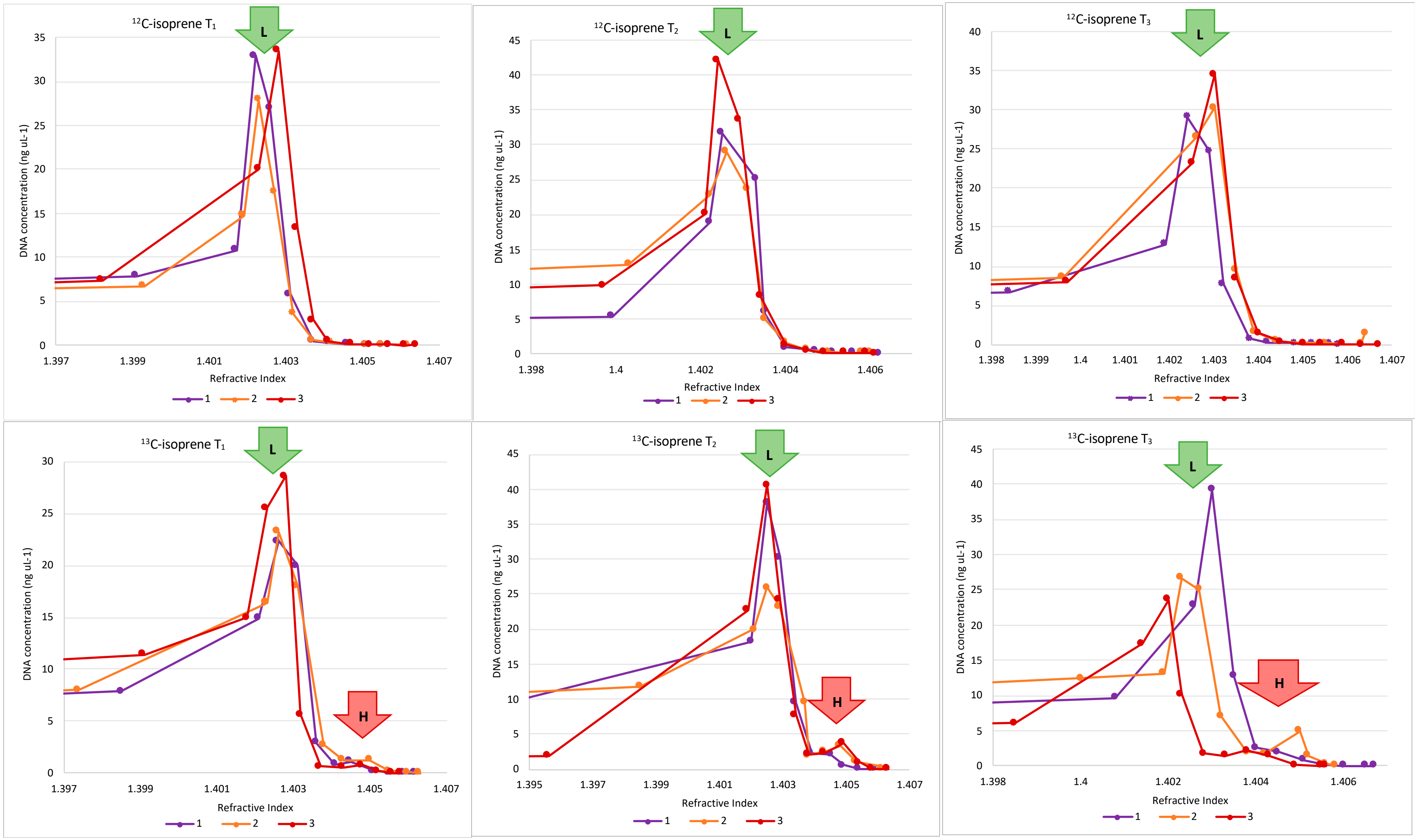

^{12}C-isoprene T_1
^{12}C-isoprene T_2
^{12}C-isoprene T_3
^{13}C-isoprene T_1
^{13}C-isoprene T_2
^{13}C-isoprene T_3
L
H
DNA concentration (ng uL-1)
Refractive Index
1
2
3

B

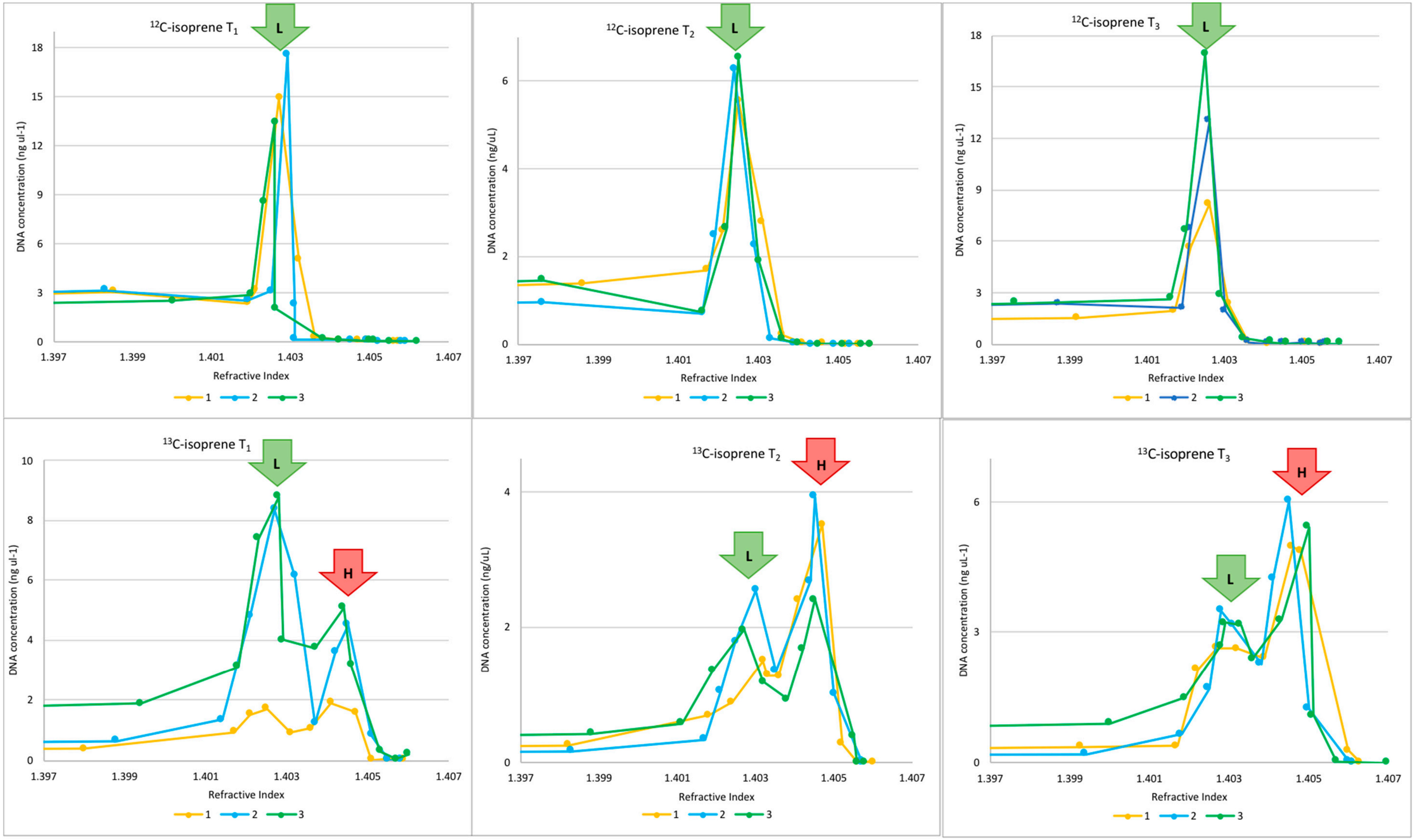

12C-isoprene T1
L
DNA concentration (ng ul-1)
Refractive Index
1
2
3
12C-isoprene T2
L
DNA concentration (ng/uL)
Refractive Index
12C-isoprene T3
L
DNA concentration (ng uL-1)
Refractive Index
13C-isoprene T1
L
H
DNA concentration (ng ul-1)
Refractive Index
13C-isoprene T2
L
H
DNA concentration (ng/uL)
Refractive Index
13C-isoprene T3
L
H
DNA concentration (ng uL-1)
Refractive Index

Figure S3. Separation ^{12}C and ^{13}C DNA of oil palm soil (A) and oil palm leaf washings (B) throughout the DNA-SIP experiments.

DNA concentration and density of CsCl fractions obtained after ultracentrifugation of extracted DNA from enriched soil (A) and leaf washings (B) microcosms after enrichment with ^{12}C-isoprene (top panel) and ^{13}C-labelled isoprene (bottom panel) for each replicate (1, 2 and 3) throughout the DNA-SIP experiments (T_1, T_2 and T_3, refer to Table S1). The CsCl fractions chosen to represent ^{13}C-labelled (heavy fraction) and unlabelled ^{12}C-DNA (light fraction) are indicated: H arrow: heavy DNA; L arrow: light DNA.

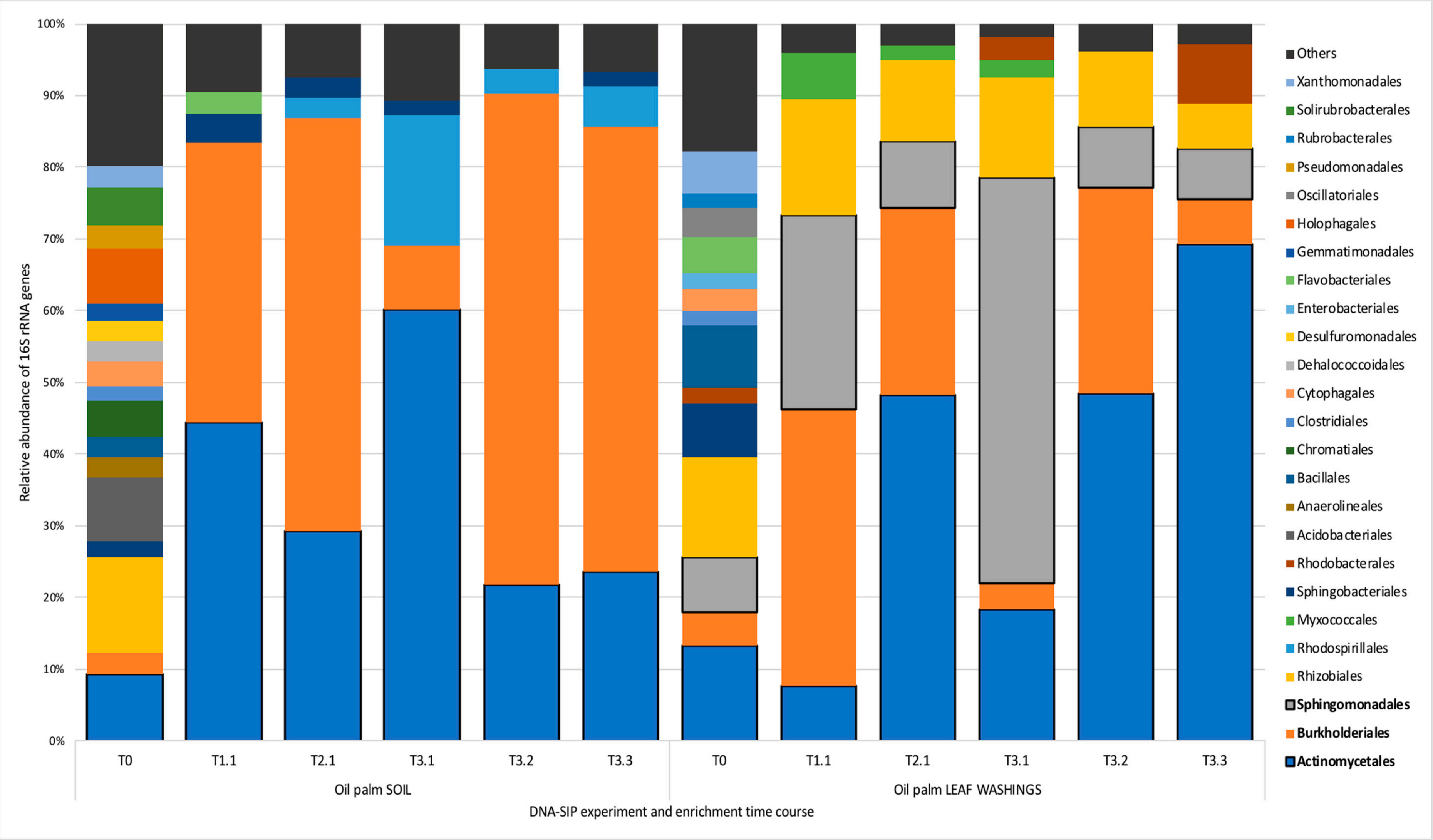

Figure S4. Relative abundance of bacterial orders during DNA-SIP enrichments

Relative abundance of bacterial 16S rRNA genes (at the order phylogenetic level) obtained by PCR of enriched and un-enriched DNA extracted from oil-palm soil and leaf-washing samples. Results include an un-enriched (un-fractionated) sample and pooled heavy DNA fractions (Figure S3) for three time points T_1, T_2 for one replicate (shown as $T_{1.1}$, $T_{2.1}$) and T_3 for all three replicates (shown as $T_{3.1}$, $T_{3.2}$ and $T_{3.3}$; refer to Table S1 for the time course). Soil samples, enriched towards the left, and leaf washings, enriched towards the right. Only 16S rRNA gene sequences with a relative abundance of greater than 2% are shown. 16S rRNA gene sequences with a relative abundance of less than 2% are grouped together as "others". Representatives from the order Actinomycetales and Sphingomonadales, highlighted with black border, were isolated from the DNA-SIP enrichments.

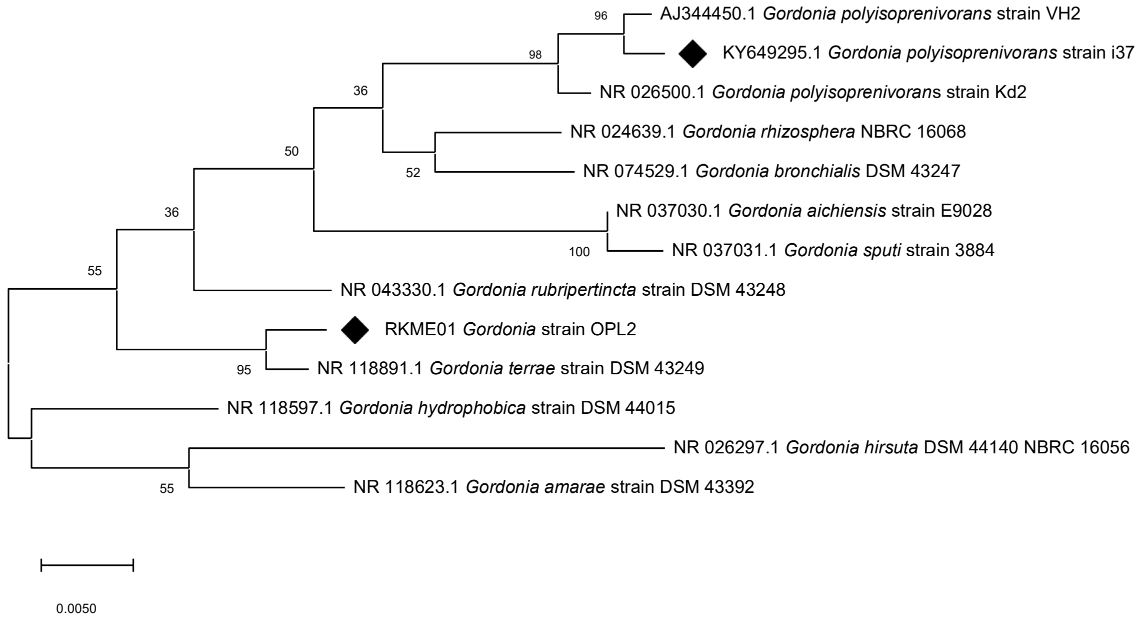

Figure S5. 16S rRNA gene phylogenetic tree of representative members of the *Gordonia* genus and the oil palm leaf isolate *Gordonia* strain OPL2.

Maximum Likelihood tree includes thirteen 16S rRNA gene sequences, twelve representatives from the *Gordonia* genus and *Gordonia* strain OPL2. Following removal of gaps and missing data, there were 1,337 bp in the alignment. Bootstrap values (1000 replications) are shown. Confirmed isoprene-degrading isolates from the *Gordonia* genus are indicated with black diamonds, including *Gordonia* strain OPL2 isolated in this study. The scale bar shows nucleotide substitutions per site.

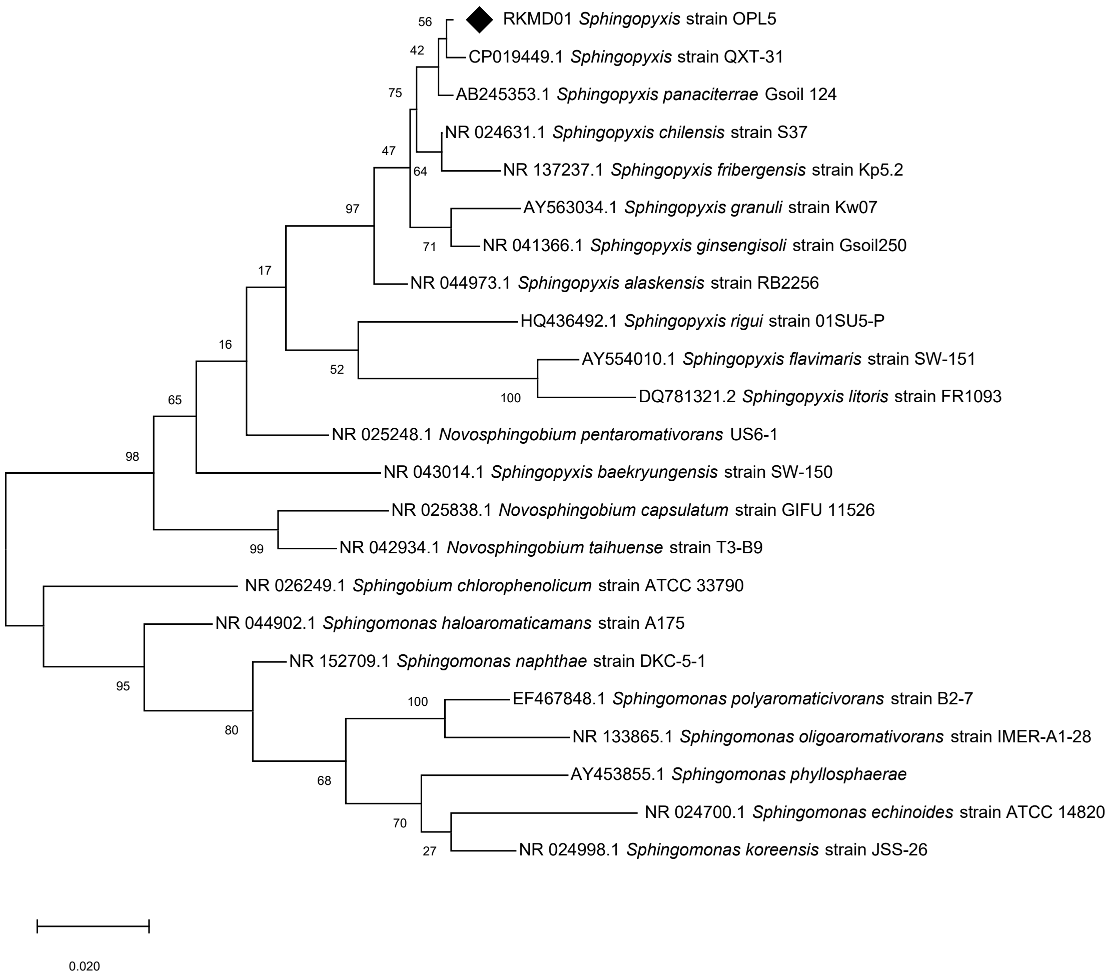

Figure S6. 16S rRNA gene phylogenetic tree of representative members of the Sphingomonadaceae family and the oil palm leaf isolate *Sphingopyxis* sp. OPL5

Maximum likelihood tree includes twenty-two sequences, twenty-one type strain representatives from the Sphingomonadaceae family and *Sphingopyxis* strain OPL5. Following removal of gaps and missing data, there were 1,370 bp in the alignment. Bootstrap percentages (1000 replications) are shown at the nodes. The strain isolated in this study is indicated with a black diamond. The scale bar shows nucleotide substitutions per site.

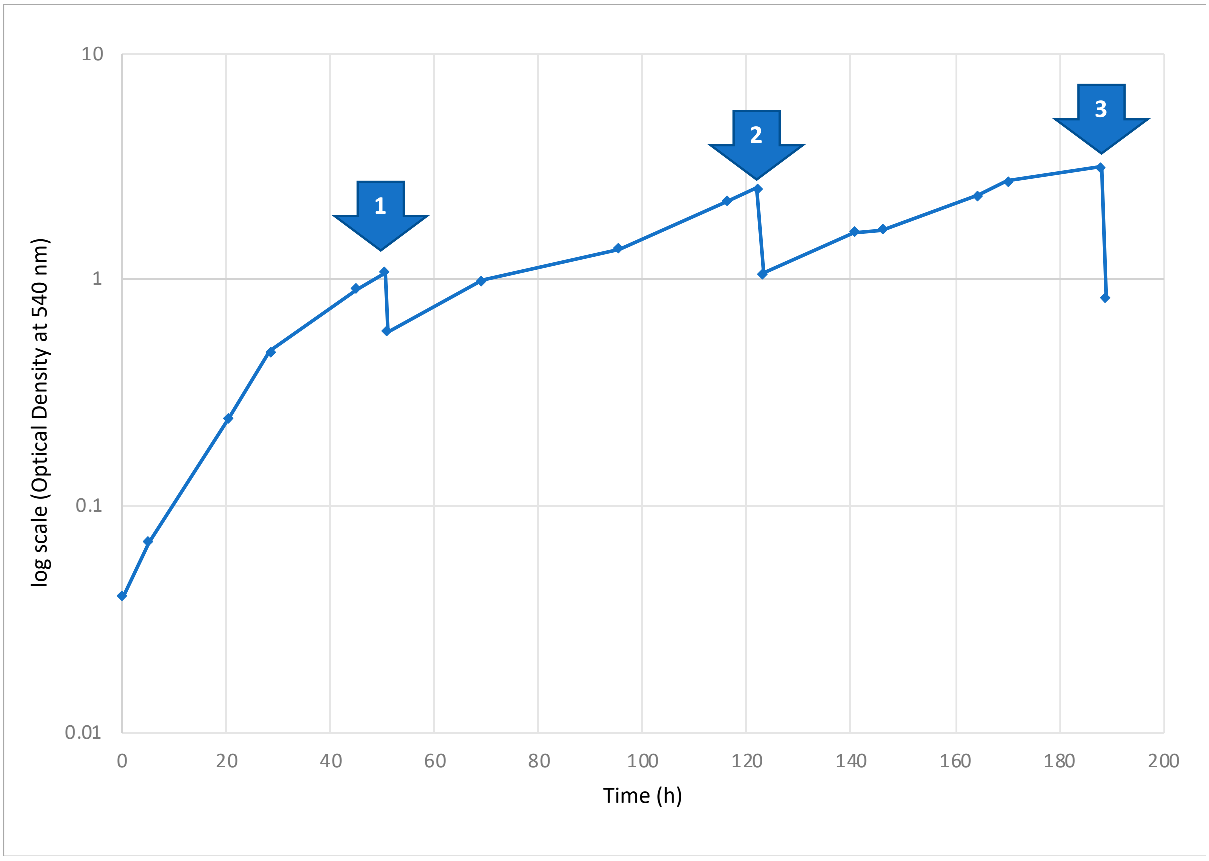

Figure S7. Growth of *Sphingopyxis* sp. OPL5 in a 4 litre fermenter with isoprene.

Sphingopyxis OPL5 cells were grown in minimal medium and supplied with isoprene vapour in air. Arrows indicate the three harvesting times.

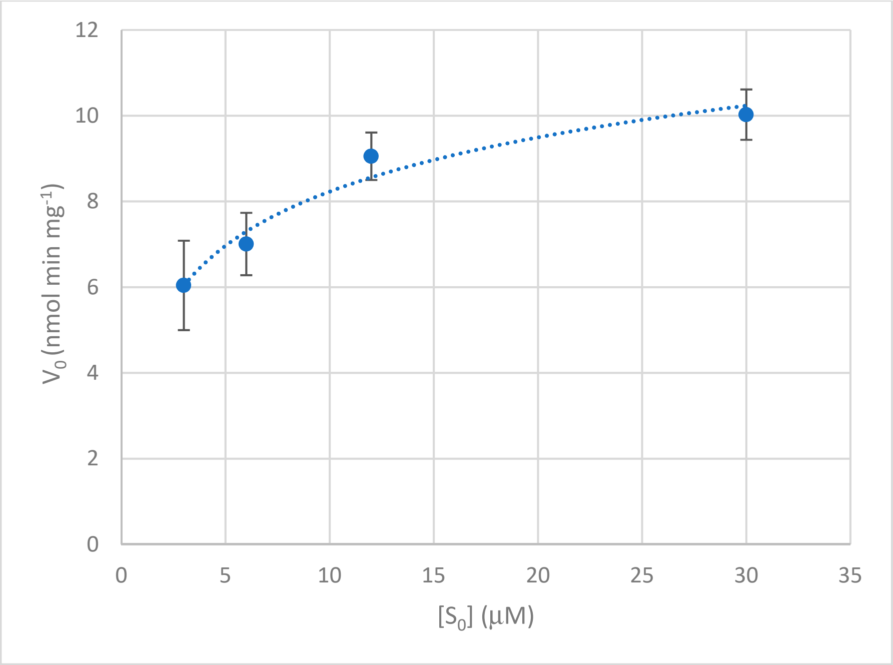

Figure S8. Rate of oxygen uptake (V_0) as a function of isoprene concentration [S_0] in solution for *Sphingopyxis* strain OPL5 cells, determined using a Clark-type oxygen electrode [12].

Table S1 Incorporation of ^{13}C-labelled carbon during DNA-SIP experiments with oil palm soil and leaf washings

Sample	Time course	^{13}C isoprene spiked into the headspace (µmol g^{-1}) *	Time (h)
OPS SIP	Native soil (T_0)	0	0
OPS SIP	Time point 1 (T_1)	31.2	142
OPS SIP	Time point 2 (T_2)	53.1	166
OPS SIP	Time point 3 (T_3)	85.9	190
OPL SIP	Native leaf washing (T_0)	0	0
OPL SIP	Time point 1 (T_1)	~25	214-226^
OPL SIP	Time point 2 (T_2)	~50	274-285.5^
OPL SIP	Time point 3 (T_3)	~75	302-311.5^

*Consumption of isoprene in DNA-SIP microcosms was monitored by gas chromatography. Incorporation of carbon was estimated assuming that 50% isoprene consumed was assimilated.

^There was variation in the consumption times and spiking of fresh isoprene for the leaf-washing replicates.

Table S2. Identity of Iso polypeptides encoded by *iso* gene clusters when compared to the corresponding Iso polypeptides from *Rhodococcus* sp. strain AD45

Isoprene-degrading isolates are in bold. wsMG designation indicates an *iso* gene-containing contig retrieved from willow soil metagenomes [2]. Percentage identities (% amino acid identity) are with respect to the Iso polypeptides of the most well-characterised isoprene-degrader *Rhodococcus* sp. AD45 [3], except for AldH1 from *Gordonia* i37 which was chosen because of its position in the cluster.

100	99-90	89-80	79-70	69-60	59-50	49-35	34-0

Isolate/contig											
***Rhodococcus* sp. AD45**	**IsoG**	**IsoH**	**IsoI**	**IsoJ**	**-**	**IsoA**	**IsoB**	**IsoC**	**IsoD**	**IsoE**	**IsoF**
***Gordonia* sp. i37**	79	79	83	71	**AldH1**	86	57	78	74	71	60
***Gordonia* sp. OPL2**	78	81	79	68	84	87	66	81	73	68	61
***Variovorax* sp. WS11**	60	48	48	55	50	73	38	40	58	53	41
***Sphingopyxis* sp. OPL5**	61	61	45	58	50	72	44	54	50	53	44
wsMG4 (*Sphingopyxis*-like)	-	-		57	49	72	44	50	56	51	42

References

1. Klindworth, A.; Pruesse, E.; Schweer, T.; Peplies, J.; Quast, C.; Horn, M.; Glöckner, F.O. Evaluation of general 16S ribosomal RNA gene PCR primers for classical and next-generation sequencing-based diversity studies. *Nucleic Acids Res.* **2013**, *41*, 1–11, doi:10.1093/nar/gks808.
2. Larke-Mejía, N.L.; Crombie, A.T.; Pratscher, J.; McGenity, T.J.; Murrell, J.C. Novel isoprene-degrading Proteobacteria from soil and leaves identified by cultivation and metagenomics analysis of stable isotope probing experiments. *Front. Microbiol.* **2019**, *10*, doi:10.3389/fmicb.2019.02700.
3. Crombie, A.T.; Khawand, M. El; Rhodius, V.A.; Fengler, K.A.; Miller, M.C.; Whited, G.M.; Mcgenity, T.J.; Murrell, J.C. Regulation of plasmid-encoded isoprene metabolism in *Rhodococcus*, a representative of an important link in the global isoprene cycle. *Environ. Microbiol.* **2015**, *17*, 3314–3329, doi:10.1111/1462-2920.12793.
